# Effects of a Chimeric Lysin against Planktonic and Sessile *Enterococcus faecalis* Hint at Potential Application in Endodontic Therapy

**DOI:** 10.3390/v10060290

**Published:** 2018-05-29

**Authors:** Wuyou Li, Hang Yang, Yujing Gong, Shujuan Wang, Yuhong Li, Hongping Wei

**Affiliations:** 1 The State Key Laboratory Breeding Base of Basic Science of Stomatology (Hubei-MOST) & Key Laboratory of Oral Biomedicine, Ministry of Education, School of Stomatology, Wuhan University, Wuhan 430079, China; 2016203040006@whu.edu.cn; 2 Key Laboratory of Special Pathogens and Biosafety, Center for Emerging Infectious Diseases, Wuhan Institute of Virology, Chinese Academy of Sciences, Wuhan 430071, China; wangsj@wh.iov.cn; 3 State Key Laboratory of Agricultural Microbiology, College of Life Science and Technology, Huazhong Agricultural University, Wuhan 430070, China; hejin@mail.hzau.edu.cn

**Keywords:** bacteriophage lysin, *Enterococcus faecalis*, bacterial biofilm, endodontic infection, calcium hydroxide

## Abstract

*Enterococcus faecalis* is a commensal opportunistic pathogen found in the intestine, mouth, and vaginal tract of humans. As an invasive pathogen in the oral cavity, *E. faecalis* is one of the leading causes of periapical endodontic lesions. However, due to the strong biofilm-forming capacity and tolerance of *E. faecalis* to conventional antibiotics and treatments, limited therapeutic options are available. In the present study, we investigated the activity of ClyR, a chimeric lysin with extended streptococcal lytic spectrum, against planktonic and sessile *E. faecalis* cells in vitro and in an ex vivo dental model. Our results showed that ClyR has robust and rapid lytic activity against multiple *E. faecalis* strains, killing >90% planktonic cells within 1 min at a concentration of 50 μg/mL. The biochemical experiments combined with microscopy analysis revealed that ClyR degrades *E. faecalis* biofilm with high efficacy in a dose-dependent manner, reducing the survival rate to <40% within biofilms after treatment with 50 μg/mL ClyR for 1 h. In the ex vivo dental model, ClyR showed a significant biofilm removal efficacy, killing >90% viable bacteria within biofilms at a low dose of 50 μg/mL, which is much better than ampicillin and similar to calcium hydroxide, the extensively used routine intracanal medicament in the treatment of endodontics and dental traumatology. The robust activity of ClyR against both planktonic and sessile *E. faecalis* suggests the potential of ClyR in treating endodontic infections caused by *E. faecalis*.

## 1. Introduction

*Enterococcus faecalis*, an opportunistic Gram-positive pathogen, is a member of the normal microorganisms of the oral cavity, intestines, and vaginal tract of human beings and animals [1]. As a common community of oral microbiota, *E. faecalis* has found to be involved in the microflora of the root canal and the periodontal pocket that do not respond well to conventional root canal therapy [2]. In most instances, *E. faecalis* can survive as biofilm in the surface of the dentinal tubule or within granules combined with other anaerobic and facultative bacteria, making refractory periapical endodontic lesions difficult to cure [3,4]. Because of the strong biofilm formation ability, which empowers bacteria enhanced resistance to antimicrobial agents [5,6], attenuated susceptibility to host immune clearance and phagocytosis [7,8], and the increased emergence of multidrug resistance isolates [9,10], *E. faecalis* now represents a great challenge to public health worldwide [11,12]. Therefore, alternatives effective against *E. faecalis* and its biofilm are urgently needed.

In recent years, lysin therapy has attracted much attention because of the highly efficient bactericidal activity in vitro and in animal infection models [13,14,15]. Lysins encoded by bacteriophages can cause a time-clocked cell lysis from within, and can also result in rapid cell wall degradation against a susceptible Gram-positive bacterium when added exogenously [16,17]. Due to their robust cell lysis capacity, lysins have also been found capable of degrading bacterial biofilms through a layer-by-layer model, for instance, PlyC is highly active against *Streptococcus pyogenes* biofilm [15], several staphylococcal lysins (such as CF-301 [18], ClyH [19], ClyF [20], phi11 endolysin [21], SAL-2 [22], Ply187AN-KSH3b [23], P128 [24] and so on) have reported to be active against *Staphylococcus aureus* biofilm in vitro and in skin infection models, lysin LySMP is capable of removing *Streptococcus suis* biofilm [25], pneumococcal lysins Cpl-1 and Cpl-7 are efficient against *Streptococcus pneumoniae* biofilm [26], and ClyR has reported to be the first lysin that is active against *Streptococcus mutans* biofilm [27]. Few lysins with significant bactericidal activity against *E. faecalis* have been developed to date, including the chimeric lysin ClyR that comprises the CHAP domain of PlyC lysin and the cell-wall binding domain of PlySs2 lysin and shows extended streptococcal lytic spectrum [28], the natural lysin LysEF-P10 [29], and the chimeric lysin EC300 [30]. However, as far as we know, no study has been done on using lysins to treat biofilms of *E. faecalis* in dental models.

In the present study, we tested the bactericidal activity of ClyR against planktonic and sessile *E. faecalis* in vitro and in an ex vivo dental model.

## 2. Materials and Methods

### 2.1. Bacterial Strains and Protein

All *E. faecalis* strains used in this study were routinely grown in brain heart infusion (BHI) broth at 37 °C, and 3% glucose was added in biofilm cultivation. A collection of clinical *E. faecalis* isolates were obtained from different patients and provided by Wuhan University Stomatological Hospital and identified by PCR-DNA sequencing analysis combined with biochemistry test using a MicroStation system (Biolog, GENIII Omnilog Combo Plus System, Hayward, CA, USA). The chimeric lysin, ClyR, was expressed in *E. coli* BL21(DE3), purified through Ni-nitrilotriacetic acid affinity chromatography and dialyzed against phosphate-buffered saline (PBS, containing 137 mM NaCl, 2.7 mM KCl, 10 mM Na_2_HPO_4_, 2 mM KH_2_PO_4_, pH 7.4) as described previously [28].

### 2.2. In Vitro Lytic Activity Assay

Bacterial cells were cultured overnight, centrifuged, and re-suspended in PBS to a final OD_600_ of 0.8–1.0 (~10^8^ colony-forming unit (CFU)/mL). Then 160 μL of each bacterial suspension was mixed with 40 μL ClyR (to a final concentration of 12.5, 25, 50, or 100 μg/mL) in a 96-well plate (Perkin-Elmer, Shanghai, China), the drop of OD_600_ in each well was monitored simultaneously by a microplate reader (Synergy H1, BioTek, Winooski, VT, USA) at 37 °C for 30 min.

To test the time- and dose-dependent killing capacity of ClyR, *E. faecalis* ATCC 51299 cells were treated either with different concentrations of ClyR (0, 0.5, 2, 5, and 25 μg/mL) for 40 min at 37 °C, or treated with 50 μg/mL ClyR for different times (0, 0.5, 1, 5, and 15 min) at 37 °C, after that, viable cell numbers after each treatment were determined by serial ten-fold dilution and plating on BHI agar. All experiments were repeated for at least three replicates.

### 2.3. MIC Determination

The MIC (minimal inhibitory concentration) of several antibiotics (vancomycin, ampicillin, daptomycin, and erythromycin) against *E. faecalis* ATCC 51299 was performed as described [31], with minor modifications. Briefly, overnight cultures of *E. faecalis* were centrifuged and re-suspended in PBS to an OD_600_ of 0.7. After dilution 10-fold with fresh cation-adjusted Mueller-Hinton broth (CAMHB) medium, 10 μL of the bacterial dilution was added to a 96-well polystyrene tissue culture plate pre-supplemented with CAMHB containing various concentrations of antibiotics (a total volume of 200 μL) and incubated statically for 24 h at 37 °C. MIC was defined as the lowest concentration of antibiotic that inhibits visible growth.

### 2.4. In Vitro Biofilm Grown Condition and Detection Assay

*E. faecalis* biofilm was cultured in BHI supplemented with 3% glucose (BHIG) according to the method described [32], with minor modifications. Briefly, overnight cultures of *E. faecalis* ATCC 51299 were centrifuged and re-suspended in PBS to a final OD_600_ of 0.7 (~10^8^ CFU/mL). An amount of 10 μL of bacterial suspension was mixed with 190 μL BHIG in a 96-well polystyrene plate (Tissue culture treated, Nest Biotech Co., Wuxi, Jiangsu, China) and incubated for 24 h at 37 °C to allow biofilm formation. After removing the planktonic phase and washing three times with PBS, biofilms were treated with 200 μL different concentrations of ClyR (0, 50, 100, and 200 μg/mL) for 1 h at 37 °C. Washing twice with PBS to remove the residual amount of antimicrobials, biofilms were fixed by 100 μL methyl alcohol for 15 min. Then, the adhered biofilms were stained with 50 μL of 0.1% (*w*/*v*) crystal violet at room temperature for 5 min. Finally, wells were washed with flowing water and solubilized with 200 μL ethanol to detect the absorbance at 595 nm. Meanwhile, in order to determine the viable cell number within each well after antimicrobial treatment, cells were recovered by adding 100 μL PBS and scratching with a sterile swab. After ultrasonication for 2 min (to fully disaggregate biofilm), samples were series diluted and plated to BHI agar.

### 2.5. Dentin Slices Preparation and Biofilm Cultivation

The caries-free single-rooted teeth used in this study were selected from orthodontic extraction approved by the ethics committee of Wuhan University (No. 2016-30, 26 February 2016). The teeth were soaked in 0.01% NaClO solution for 6 days, shaped to a size of 4 × 4 × 2 mm semicylindrical dentin slice, and ultrasoniced for 4 min respectively with 5.25% NaClO and 6% citric acid (Sigma, Shanghai, China) at 40,000 Hz by an ultrasonic bath (SB-5200 DTD, SCIENTZ, Ningbo, China) to remove the smear layer on both sides of the specimen. Finally, all dentin slices were washed with sterile water and sterilized for 20 min at 121 °C. *E. faecalis* ATCC 51299 was cultured in BHIG in 24-well plates containing one dentin slice for 24 h to allow biofilm formation. After washing twice with PBS, the dentin slice was transferred to a sterilized tube and treated with either 50 μg/mL ClyR, 50 mg/mL ampicillin, or 0.5% calcium hydroxide (Ca(OH)_2_) for 1 h at 37 °C. Washed twice with PBS, dentin slices were ultrasoniced 2 min in PBS (to fully disaggregate biofilm), and series diluted for checking viable cell numbers on BHI agar.

### 2.6. Transmission Electron Microscopy

Overnight cultures of *E. faecalis* ATCC 51299 cells were washed with PBS and treated with different concentrations of ClyR (0, 50, 100, and 200 μg/mL) for 1 h at 37 °C. After adding glutaraldehyde to a final concentration of 2.5%, bacterial suspension was sampled and analyzed by using a transmission electron microscope (Tecnai G^2^ 20 Twin; Fei, Hillsboro, OR, USA).

### 2.7. Scanning Electron Microscopy

In order to visualize the structural changes of *E. faecalis* biofilms after exposure to ClyR, *E. faecalis* ATCC 51299 biofilms grown on glass coverslips for 24 h were washed twice with PBS (to remove the planktonic cells), and treated with 50 μg/mL ClyR for 1 h. Biofilms were then washed twice with PBS, fixed with 2.5% glutaraldehyde overnight at 4 °C, dehydrated by granted ethanol (from 30% to 100%), and analyzed by a SU8010 scanning electron microscope (SEM, Hitachi, Tokyo, Japan). The PBS treated groups were used as controls. Images were taken at ×20,000 magnification under the same instrument conditions.

### 2.8. Confocal Laser Scanning Microscopy

*E. faecalis* biofilms cultured in glass bottom dish (NEST) for 24 h were treated with various concentrations of ClyR (0, 100, and 200 μg/mL), washed twice with PBS, and stained with LIVE/DEAD BacLight bacterial viability kit (L7010, Invitrogen, Carlsbad, CA, USA) for 15 min at room temperature. Finally, biofilms were washed by distilled water, and imaged by using an UltraVIEW VoX confocal laser scanning microscope (CLSM, PerkinElmer, Waltham, MA, USA). All acquired images were analyzed by the Volocity (version 6.3.0, PerkinElmer, Waltham, MA, USA) software supplied with the instrument.

## 3. Results

### 3.1. ClyR Is Highly Active against Multiple Planktonic E. faecalis

**Figure 1:**
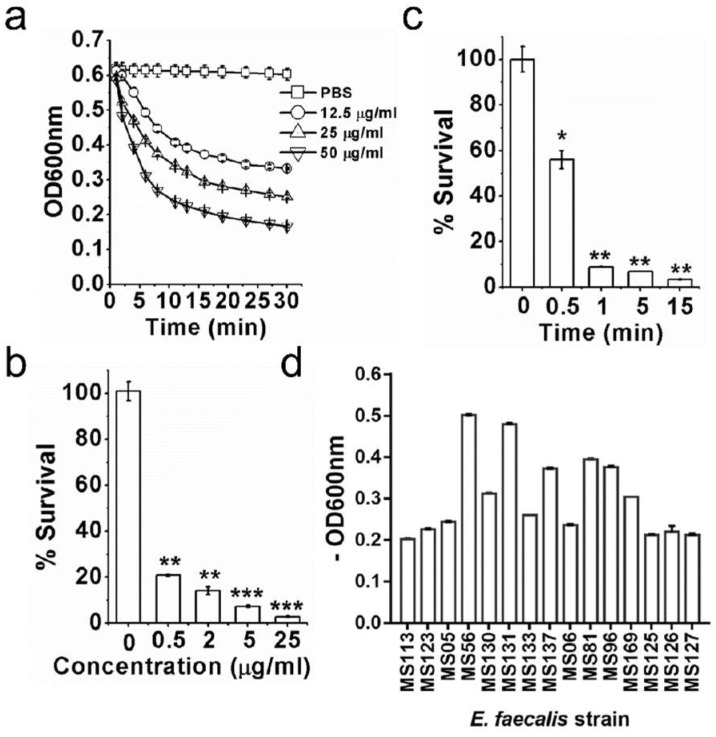
Robust and rapid bactericidal activity of ClyR against planktonic *E. faecalis*. (**a**) Lysis curves of ClyR against *E. faecalis* ATCC 51299. Bacterial cells are washed with phosphate-buffered saline (PBS) and mixed with various concentrations of ClyR (0, 12.5, 25, and 50 μg/mL), the decrease of OD_600_ is monitored by a microplate reader for 30 min at 37 °C. (**b**) Dose-dependent killing efficacy of ClyR against *E. faecalis* ATCC51299. Bacterial cells are washed with PBS and treated with various concentrations of ClyR (0, 0.5, 2, 5, and 25 μg/mL) for 40 min at 37 °C, the viable cell number after each treatment is determined by plating on brain heart infusion (BHI) agar. (**c**) Time-dependent killing efficacy of ClyR against *E. faecalis* ATCC51299. Bacterial cells are washed with PBS and treated with 50 μg/mL of ClyR at 37 °C for different times (0, 0.5, 1, 5, and 15 min), the viable cell number after each treatment is determined by plating on BHI agar. (**d**) Susceptibility of multiple *E. faecalis* strains to ClyR. The result of each treatment is compared with that of the PBS-treated controls by Two-tailed Student’s *t* test. Data is shown as mean ± standard deviation, and * *p* < 0.05; ** *p* < 0.01; *** *p* < 0.001.

In previous studies, we have confirmed that ClyR is active against one *E. faecalis* strain [28]. Herein, we examined the bacteriocidal activity of ClyR against *E. faecalis* in detail using more different isolates. As shown in Figure 1a, ClyR caused a rapid decrease in turbidity of *E. faecalis* in a dose-dependent manner, with a reduction from 0.6 (~1.36 × 10^7^ CFU/mL) to ~0.3 (~9.5 × 10^6^ CFU/mL) within 5 min under a concentration of 50 μg/mL. The dose-dependent experiments showed that 0.5 μg/mL ClyR kills ~80% viable bacteria after treatment for 40 min, and <10% survival rate is observed when treated with 5 μg/mL ClyR (Figure 1b). Moreover, treatment with 50 μg/mL ClyR for only 0.5 min eradicated ~50% viable bacteria, and >90% bacterial cells were killed after exposure to ClyR for 1 min (Figure 1c), suggesting a robust and rapid bactericidal activity. We further assessed the susceptibility of another 15 *E. faecalis* strains to ClyR, and results showed that ClyR is highly active against all strains tested (Figure 1d), with decreases in OD_600_ from 0.2 to ~0.5.

**Figure 2:**
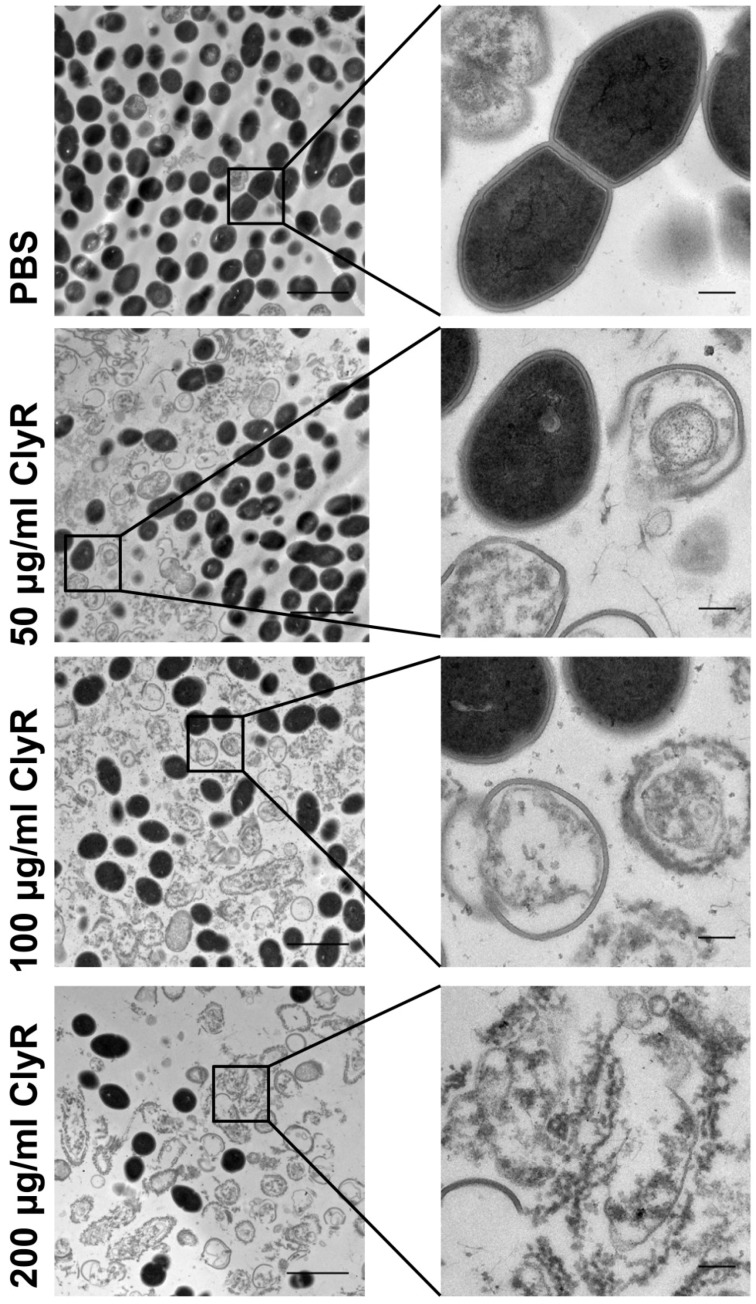
Morphology of *E. faecalis* after exposure to ClyR. *E. faecalis* ATCC 51299 cells are washed with PBS and treated with various concentrations of ClyR (0, 50, 100, and 200 μg/mL) for 1 h at room temperature. The morphology of bacterial cells after each treatment is observed by TEM. Scale bars: left panel: 2 μm; right panel: 20 nm.

In order to know the morphological change of *E. faecalis* after exposure to ClyR, we investigated the integrity of *E. faecalis* cells after treatment with various concentrations of ClyR by TEM. Results showed that the ratio of the number of cells with intact cell wall to the number of ruptured cells declined along with increasing concentration of ClyR (Figure 2, left panels). The inserted figures clearly showed that native *E. faecalis* bacteria have a holonomic and uniform cell wall, while the bacteria after treatment with ClyR exhibit destructed and heavily deformed cell morphology, resulting in the presence of many “ghost cells” (Figure 2, right panels). This observation is quite consistent with the phenomenon of lysin-mediated osmotic cell lysis reported elsewhere [33].

### 3.2. ClyR Is Active against E. faecalis Biofilm In Vitro

**Figure 3:**
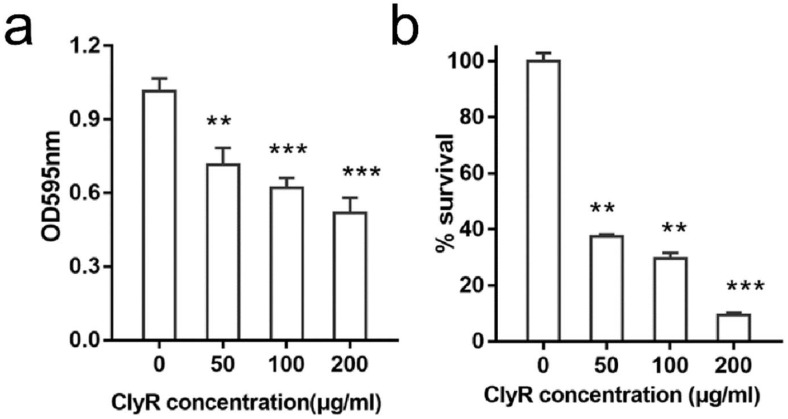
High activity of ClyR against *E. faecalis* biofilm. (**a**) Crystal violet staining analysis of *E. faecalis* biofilms. *E. faecalis* ATCC 51299 cells are cultured in BHI supplemented with 3% glucose (BHIG) for 24 h to allow biofilm formation, after washing with PBS, biofilms are treated with various concentrations of ClyR (0, 50, 100, and 200 μg/mL) at 37 °C for 1 h, residual biofilms are stained with 0.1% crystal violet and detected at OD_595_ by a microplate reader after resolved by ethanol. (**b**) Survival rate of *E. faecalis* biofilms after treatment with ClyR. 24 h-aged *E. faecalis* ATCC 51299 biofilms are treated with various concentrations of ClyR (0, 50, 100, and 200 μg/mL) at 37 °C for 1 h, the viable cells after each treatment is determined and compared with that of PBS treated controls by Two-tailed Student’s t test. Data is shown as mean ± standard deviation, and ** *p* < 0.01; *** *p* < 0.001.

Next, we assessed the degradation efficacy of ClyR against established *E. faecalis* biofilms. The crystal violet staining assay showed that ClyR causes a significant dissociation of mature biofilms in a dose-dependent manner (Figure 3a). Consequently, the number of viable bacteria recovered from biofilms decreased along with the increased dose of ClyR (Figure 3b), with less than 40% (from 2.17 × 10^7^ CFU/mL to 8.2 × 10^6^ CFU/mL) and ~10% (from 2.17 × 10^7^ CFU/mL to 2.2 × 10^6^ CFU/mL) survival after treatment with 50 μg/mL and 200 μg/mL ClyR for 1 h, respectively.

### 3.3. Microscopy Analysis of E. faecalis Biofilms after Exposure to ClyR In Vitro

**Figure 4:**
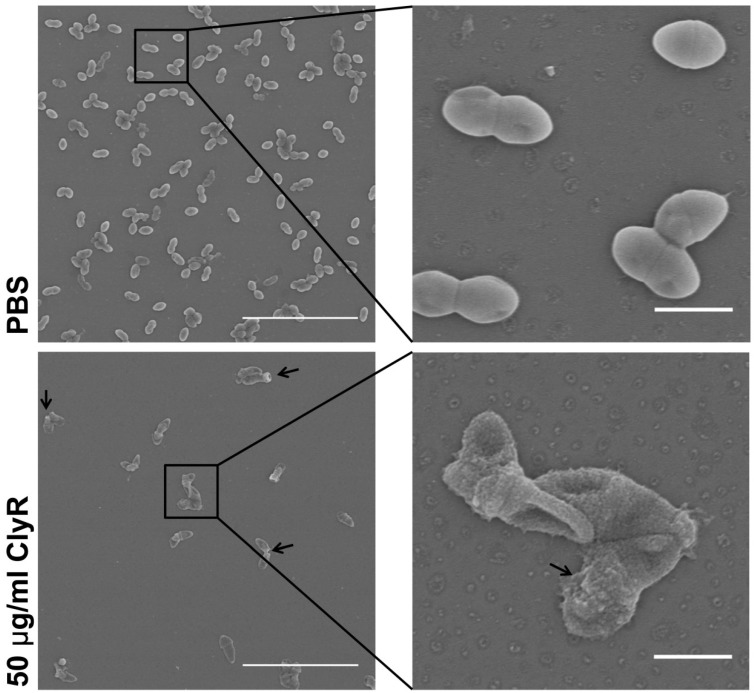
SEM analysis of *E. faecalis* biofilms after exposure to ClyR. *E. faecalis* ATCC 51299 is cultured in BHIG on glass coverslips for 24 h to allow biofilms, after washing with PBS, biofilms are treated with 50 μg/mL ClyR for 1 h at 37 °C, the residual biofilms are sampled and analyzed by SEM. PBS treated groups are used as controls. Scale bars: left panel: 10 μm; right panel: 1 μm.

In order to investigate the mechanism and the dynamics of ClyR against *E. faecalis* biofilms, we analyzed the phenotypic morphology and structure of *E. faecalis* biofilms after exposure to ClyR. The SEM images showed that ClyR causes a direct cell lysis within biofilms and thus gives rise to the rapid degradation of mature biofilm, displaying rough and shrink cell surfaces (Figure 4, arrow). By contrast, native *E. faecalis* bacteria within biofilms exhibited a smooth and full morphological character (Figure 4, upper).

**Figure 5:**
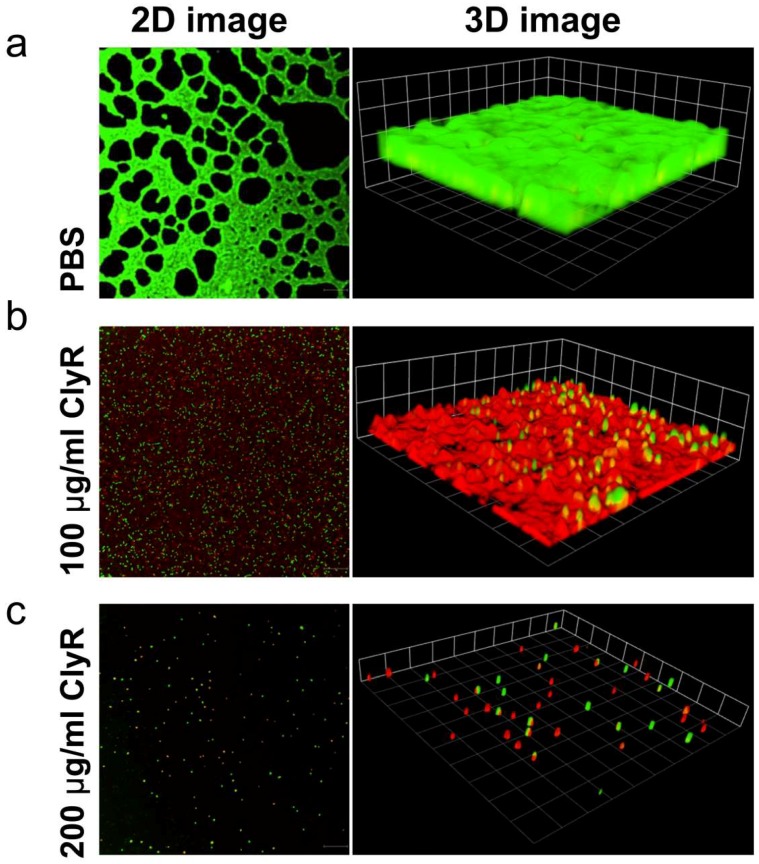
Confocal laser scanning microscope (CLSM) analysis of *E. faecalis* biofilms after exposure to 0 (**a**), 100 μg/mL (**b**) or 200 μg/mL (**c**) ClyR. *E. faecalis* ATCC 51299 biofilms grown for 24 h are treated with different concentrations of ClyR (0, 100 and 200 μg/mL) for 1 h at 37 °C. After washing with PBS, biofilms are stained with LIVE/DEAD reagent and analyzed by confocal laser scanning microscopy. Scale bars: 30 μm

Further, we observed the structure of *E. faecalis* biofilms by using LIVE/DEAD dye, by which the living bacteria within biofilms were labeled as green, while the dead bacteria are labelled as red. As shown in Figure 5a, *E. faecalis* biofilms cultured for 24 h showed heavy but heterogeneous biofilm layers showing bright green fluorescence. After treatment with 100 μg/mL ClyR for 1 h, a large number of red spots, representing dead bacterial cells, were observed within biofilms (Figure 5b, left), showing a weakened biofilm with many dead but still adhered cells (Figure 5b, right). However, in *E. faecalis* biofilms after treatment with 200 μg/mL ClyR, few viable bacteria could be found, showing a scattered and residual biofilm with few live and dead cells adhered (Figure 5c). These results suggested that ClyR causes death and dysfunction to *E. faecalis* bacteria within biofilms through direct cell lysis, and then the heterogenization and exfoliation of bacterial biofilms, and finally the formation of degraded biofilms with few adhered bacterial cells.

### 3.4. Ex Vivo Dentin Slice Biofilm Model

**Figure 6:**
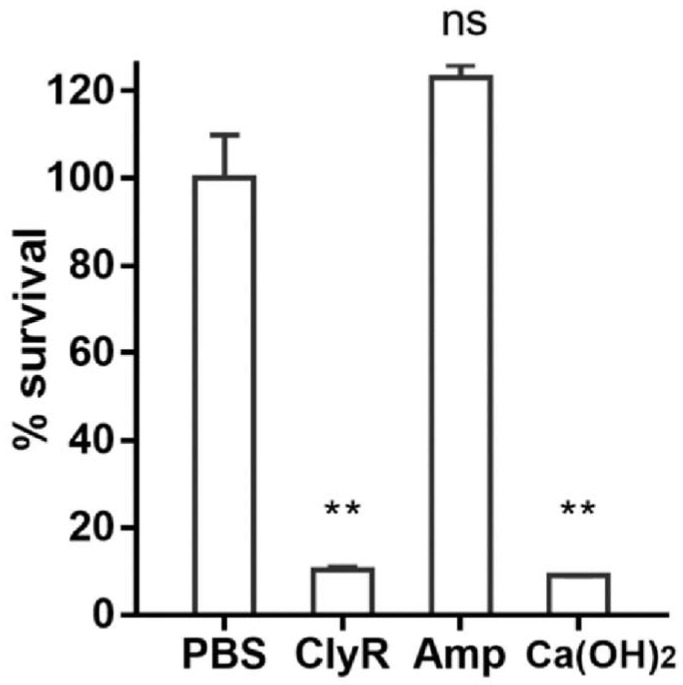
Robust biofilm removal efficacy of ClyR in an ex vivo dental model. *E. faecalis* ATCC 51299 biofilms grown on dentin slices are treated either with 50 μg/mL ClyR, 50 mg/mL ampicillin, or 0.5% Ca(OH)_2_ for 1 h at 37 °C, after washing with PBS, viable cell within biofilm after each treatment is recovered by plating on BHI agar, and compared with that of PBS treated controls by Two-tailed Student’s *t* test. Data is shown as mean ± standard deviation, and ns: not significant; ** *p* < 0.01.

Since *E. faecalis* is a major pathogen responsible for refractory periapical periodontitis, we further compared the anti-biofilm capacity of ClyR in an ex vivo dentin slice biofilm model with that of calcium hydroxide (Ca(OH)_2_), a chemical reagent that is commercially used for the treatment of a variety of dental pulp and periapical diseases [34,35,36]. *E. faecalis* ATCC 51299 is highly resistant to vancomycin (MIC = 32 μg/mL) but susceptible to ampicillin (MIC = 2 μg/mL, Table S1), therefore, we took ampicillin into comparison. Results showed that a high concentration of ampicillin of up to 50 mg/mL (25,000-fold of MIC) did not show any biofilm removal efficacy (Figure 6). However, a low concentration of ClyR (50 μg/mL) killed >90% viable bacteria within biofilms within 1 h, comparable to that of a high concentration of Ca(OH)_2_ (Figure 6).

## 4. Discussion

*E. faecalis* is a commensal opportunistic pathogen in the intestines of humans and animals and can also be found in the mouth and vaginal tract [37]. Under certain conditions, it can spread to other sites of body and cause invasive infections, such as neonatal sepsis, peritonitis, wound and urinary tract infections, and even life-threatening nosocomial infections [38]. However, due to its intrinsic resistance to multiple first-line antimicrobials [39] and easy access to acquiring enhanced resistance by forming biofilm or hosting horizontal transferred antimicrobial genes [40], treatment of an infection caused by *E. faecalis* is difficult, with limited therapeutic options [41]. In the present study, we reported that a chimeric lysin, ClyR, has high bacteriocidal activity against multiple planktonic *E. faecalis* strains, and exhibits good efficacy in removing *E. faecalis* biofilms in vitro, providing a potential alternative to treat *E. faecalis* infections.

ClyR is a chimeric lysin with a broad bactericidal spectrum, based on our previous studies, including *S. pyogenes*, *S. agalactiae*, *S. dysgalactiae*, *S. mutans*, *S. pneumoniae*, *S. suis*, *S. aureus*, and *E. faecalis* [28]. We further demonstrated here that ClyR is highly active against multiple planktonic *E. faecalis* strains (Figure 1d), including vancomycin-resistant strain ATCC 51299, killing >90% bacterial cells within only 1 min (Figure 1c), and the effective concentration of ClyR is low to 0.5 μg/mL (Figure 1b). Because the killing mechanism of ClyR is based on the direct digestion of bacterial peptidoglycan linkages and thus the osmotic cell lysis (Figure 2), absolutely differing from that of conventional antibiotics, the possibility that *E. faecalis* may gain resistance to ClyR is low, similar to the observations reported by other researchers that bacterial strains maintain susceptibility to lysins after continuous exposure [42,43]. Taking into account the robust tolerance of ClyR to various environmental factors, including a broad suitable pH range of 5–11, unsusceptible to EDTA (high up to 50 mM) and NaCl (up to 1000 mM), good protection in the animal infection model [28], and low cytotoxicity [27], ClyR may represent a good candidate for treating infections caused by multidrug resistant *E. faecalis*.

Our data also showed that ClyR has high anti-biofilm activity against *E. faecalis* in a dose-dependent manner, with <40% survival rate observed within *E. faecalis* biofilms cultured under rich nutrition conditions after treatment with 50 μg/mL ClyR for 1 h (Figure 3). Further analysis revealed that ClyR causes an obvious deformation and destruction of bacterial cells within biofilms through direct peptidoglycan hydrolysis (Figure 4), a phenomenon similar to the action of ClyH against *S. aureus* biofilms [19]. The fluorescence microscopy analysis clearly illustrated that the structural integrity and homogeneity of *E. faecalis* biofilms decline gradually with the increased dose of ClyR (Figure 5). Interestingly, large amounts of dead cells remain adhered and involved in biofilms after treatment with a middle dose of ClyR (100 μg/mL, Figure 5b), while only a few dead cells were present in the scattered biofilm after treatment with a high dose of ClyR (200 μg/mL, Figure 5c), suggesting ClyR degrades *E. faecalis* biofilm in a layer-by-layer manner, similar to the mechanism involved in PlyC’s activity against *S. pyogenes* biofilms [15]. As far as we know, this is the first time the interaction between a lysin and *E. faecalis* biofilm in vitro has been reported. Although the interaction dynamics between lysins and bacterial biofilms in the site of an infection are, to date, still far from revealed, our present data supports new insight into the mechanism a lysin with extended lytic spectrum utilized to kill sessile *E. faecalis*.

*E. faecalis* is also one of the leading causes of periapical endodontic lesions due to its strong tolerance and biofilm-forming capacity that can survive the changing pH, temperature, and osmotic pressure in the oral cavity. Currently, few options are available for the treatment of refractory periapical periodontitis caused by *E. faecalis* [3,4]. Ca(OH)_2_ has been used extensively as a routine intracanal medicament to disinfect the entire root canal system in the treatment of endodontics and dental traumatology [16,44]. Therefore, in the present study, we compared the anti-biofilm capacity of ClyR against *E. faecalis* biofilm formed in dentine slices with that of Ca(OH)_2_ and ampicillin. Encouragingly, ClyR showed a significantly high biofilm removal efficacy, killing >90% viable bacterial cells within biofilms at a low dose of 50 μg/mL, much better than 25,000× MIC ampicillin, but similar to Ca(OH)_2_, (Figure 6), suggesting it as a potent alternative in endodontic therapy associated with *E. faecalis*.

Compared with Ca(OH)_2,_ there are several advantages to introducing ClyR into endodontic applications although its in vivo anti-biofilm activity still needs to be established. On one hand, ClyR will only eliminate susceptible bacteria without disturbing other microbes involved in the oral microbiota, and the risk of resistance generation is low. However, many bacterial species recovered from the microbiota with refractory apical periodontitis already have resistance to Ca(OH)_2_ [2,45], and are thus responsible for many endodontic failures [4]. On the other hand, ClyR is proteinaceous with rare cytotoxicity and stimulation to the host immunity. Meanwhile, Ca(OH)_2_ has multiple potential side effects, including the formation of calcite granulations [46], reduced flexural strength and lower fracture resistance to dentine [47,48], calcium ions diffusion from Ca(OH)_2_-containing materials in endodontically-treated teeth [49], and detrimental effects on periodontal tissues and host immunity [50,51,52]. Taking the good stability and rapid bactericidal capacity of ClyR into consideration, one can easily find that ClyR represents a potential alternative to Ca(OH)_2_ in the management of endodontic problems.

Our previous study has reported the anti-biofilm activity of ClyR against *S. mutans*, the major pathogen for caries, in vitro and in a mouse colonization model [27]. One important finding was that ClyR significantly reduces the colonization of *S. mutans* in the oral cavity without eliciting specific antibodies. Together with our present data showing high lytic activity of ClyR against *E. faecalis* and its biofilm, our researches highlight the potent application of ClyR in treating dental-associated diseases in oral environments. One does not need to worry about the effects of antibodies and thus derived concerns on the host immunity, a propriety that is of special importance in the development and transformation of lysin-derived therapies.

In conclusion, we report here the high activity of ClyR, a chimeric lysin with extended streptococcal lytic spectrum, against planktonic and sessile *E. faecalis* cells in vitro and in an ex vivo dental model. The robust bactericidal activity of ClyR against *E. faecalis*, together with the reported good protective efficacy in the infection model and high activity against *S. mutans* biofilms, suggests that ClyR may be a good alternative in treating infection caused by *E. faecalis*, especially in the oral environment.

## Supplementary Materials

Supplementary materials can be found at http://www.mdpi.com/1999-4915/10/6/290/s1.
