# Supplementary material for: Effects of a Chimeric Lysin against Planktonic and Sessile Enterococcus faecalis Hint at Potential Application in Endodontic Therapy
Source: Viruses. 2018 May 29;10(6):290. doi: 10.3390/v10060290 (PMC6024690; doi:10.3390/v10060290)
Supplement: Supplementary file 1 [file viruses-10-00290-s001.pdf]

Supplementary materials

Effects of a chimeric lysin against planktonic and sessile *Enterococcus faecalis* hint potential application in endodontic therapy

Wuyou Li[1,#], Hang Yang[2,#,*], Yujing Gong[3], Shujuan Wang[2], Yuhong Li[1,*], Hongping Wei[2,*]

Table S1. Susceptibilities of *E. faecalis* ATCC 51299 to various antibiotics.

Strain	MIC (μg/ml)			
	Ampicillin	Vancomycin	Daptomycin	Erythromycin
E. faecalis ATCC 51299	2	32	>64	>16
